# Healable Anti-Corrosive and Wear-Resistant Silicone-Oil-Impregnated Porous Oxide Layer of Aluminum Alloy by Plasma Electrolytic Oxidation

**DOI:** 10.3390/nano13182582

**Published:** 2023-09-18

**Authors:** Yeji Shin, Kichang Bae, Sumin Lee, Hweeyong Kim, Dongmin Shin, Donghyun Kim, Eunyoung Choi, Hyoung-Seok Moon, Junghoon Lee

**Affiliations:** 1 Department of Metallurgical Engineering, Pukyong National University, Busan 48513, Republic of Korea; yeahhji_s98@pukyong.ac.kr (Y.S.); kichbae@pknu.ac.kr (K.B.); smlee7@pukyong.ac.kr (S.L.); quddkfl85@pukyong.ac.kr (H.K.); shin2202@pukyong.ac.kr (D.S.); 2 Korea Institute of Ceramic Engineering and Technology, Jinju 52851, Republic of Korea; dhkim1208@kicet.re.kr; 3 Korea Institute of Industrial Technology, Busan 46938, Republic of Korea; cossud@kitech.re.kr (E.C.); hyoungseok.moon@kitech.re.kr (H.-S.M.)

**Keywords:** plasma electrolytic oxidation, oil impregnation, silicone oil, corrosion resistance, wear resistance

## Abstract

Lubricant (or oil)-impregnated porous surface has been considered as a promising surface treatment to realize multifunctionality. In this study, silicone oil was impregnated into a hard porous oxide layer created by the plasma electrolytic oxidation (PEO) of aluminum (Al) alloys. The monolayer of polydimethylsiloxane (PDMS) from silicone oil is formed on a porous oxide layer; thus, a water-repellent slippery oil-impregnated surface is realized on Al alloy, showing a low contact angle hysteresis of less than 5°. This water repellency significantly enhanced the corrosion resistance by more than four orders of magnitude compared to that of the PEO-treated Al alloy without silicone oil impregnation. The silicone oil within the porous oxide layer also provides a lubricating effect to improve wear resistance by reducing friction coefficients from ~0.6 to ~0.1. In addition, because the PDMS monolayer can be restored by frictional heat, the water-repellent surface is tolerant to physical damage to the oxide surface. Hence, the results of this fundamental study provide a new approach for the post-treatment of PEO for Al alloys.

## 1. Introduction

Plasma electrolytic oxidation (PEO) is a surface treatment technology that forms a very stable porous oxide film on base metals using a discharge phenomenon in the electrolyte [1]. A high voltage induces a spark or arc plasma discharge, creating a local high-temperature spot in the electrolyte, enabling oxidation of the metal and the rapid melting and solidification of the oxides [2,3,4]. Because the oxide layer formed by PEO is very hard and chemically stable, it is used for the surface treatment of highly active metals, such as aluminum (Al) and magnesium (Mg) [5]. In particular, PEO treatment of Al alloys can form high-hardness oxide layers that cannot be obtained via conventional anodization [6], thereby extending the application fields of PEO-treated Al alloys to oil/gas extraction, the biomedical field, the aerospace industry, and automotive engines [2,7]. However, owing to the extremely high temperatures generated by the discharge in the oxide layer and rapid solidification of molten oxide in the electrolyte, microcracks and micropores are inevitably formed in the PEO oxide layer [8]. The micropores are formed through the release of gas during the oxidation, and microcracks are formed due to intense thermal stress resulting from the rapid cooling of the molten oxide when in contact with the surrounding cold electrolytes [9,10]. Therefore, such micropores and microcracks are inevitably formed during PEO treatment. These cracks and pores provide penetration pathways for corrosive media toward the metal substrate; therefore, the PEO layer does not consistently show excellent corrosion resistance [11,12]. In addition, owing to the brittle and porous nature of the oxide layers formed by PEO, the surface has poor wear resistance, even though it has high hardness [8,13,14]. Therefore, various post-treatment processes for alleviating defects (e.g., cracks and pores) in the PEO layer have been reported, such as cerium-based sealing treatment [15,16], sol-gel treatment [17], and polytetrafluoroethylene (PTFE) nanoparticle coating [18]. Nevertheless, an effective post-treatment process that simultaneously improves both the corrosion resistance and wear resistance of the PEO layer has been rarely introduced.

Meanwhile, porous surface structures impregnated with immiscible lubricants (i.e., slippery liquid-infused porous surface, SLIPS) have been in the spotlight as a multifunctional surface treatment with the capacity to solve various shortcomings of solid surfaces. The liquid lubricant impregnated within the porous structure inhibits contact between external matter and the surface, while the porous structure immobilizes the liquid lubricant to prevent drain-out [19]. Therefore, the lubricant-impregnated porous surfaces exhibit various functionalities, such as omniphobicity [20], corrosion resistance [21], drag-reduction [22,23], anti-/de-icing [24,25,26], anti-biofouling [27,28,29], and self-healing ability [30,31]. Generally, perfluorinated lubricants that are immiscible with a wide range of liquids have been used for the fabrication of multifunctional lubricant-impregnated surfaces [32,33]. A hydrophobizing coating with PTFE, perfluorooctyltriethoxysilane (PTES), perfluorodecyltriethoxysilane (FDTS), or octadecyltrichlorosilane (OTS) is employed to obtain a chemical affinity between the perfluorinated lubricant and the porous structure, which is critical for the fabrication of de-wettable lubricant-impregnated surfaces [34] because the formation of an additional layer on the porous structure changes the disjoining pressure affecting the stability of liquid film [35,36]. In cases using hydrocarbon lubricants, the hydrophobizing coating (e.g., octadecylichlorosilane (OTS), PTFE, FDTS) of porous surface structures is also necessary. Without these surface coatings of hydrocarbon, the external liquid can come into contact with the solid structure; thus, the exceptional de-wetting performance and multi-functionalities cannot be realized. However, despite their importance, these hydrophobizing coatings can be easily damaged by physical impact and contact, and the damaged area possibly loses its original performance [37]. A silicone-oil-impregnated surface was introduced to overcome these limitations. Because silicone oil forms a self-assembled monolayer (SAM) of polydimethylsiloxane (PDMS) on the oxide surface, the surface automatically obtains a good chemical affinity to silicone oil without an additional coating process [38,39,40]. Moreover, the formation of a PDMS-SAM layer on the oxide surface can be accelerated by ultraviolet irradiation or heating. This indicates that the PDMS functional layer improving the chemical affinity with the lubricant can be regenerated as long as silicone oil is retained within the porous oxide layer [40,41]. Moreover, silicone-oil-impregnated surfaces also can exhibit multifunctional properties similar to those of perfluorinated lubricant-impregnated surfaces [42].

In this study, we employed silicone oil impregnation as a post-treatment of PEO of Al alloy to improve the corrosion resistance and wear resistance. The silicone oil impregnated in the cracks and pores of the oxide layer formed by PEO is expected to inhibit not only the penetration of corrosive media to the Al substrate but also the contact of liquid to the oxide surface. Moreover, the silicone oil impregnated within the oxide layer provides lubrication between the oxide surface and other solid matter, thereby enhancing wear resistance. Therefore, experimental studies are systematically performed to demonstrate the efficacy of the combination of PEO and silicone oil impregnation for the enhancement of corrosion and wear resistance of the Al alloy.

## 2. Materials and Methods

An Al alloy 6061 plate (thickness: 2 mm) cut into a size of 30 × 30 mm^2^ was used as a substrate for surface treatment. Before PEO treatment, the specimen is degreased and activated in a 10 wt.% NaOH solution at 80 °C for 5 min. An aqueous solution of 10 g/L of Na_2_SiO_3_ and 4 g/L of NaOH was used as the PEO electrolyte and maintained at a temperature of 10 °C using a circulation cooling system. An AISI 304 stainless steel plate of 30 × 100 mm^2^ (thickness: 1 mm) was used as the cathode. During the PEO treatment, an anodic current density of 100 mA/cm^2^ was applied to the Al alloy plate for 10, 20, 30, and 40 min. After PEO treatment, the sample was washed with tap water, and then dried with compressed air.

In total, 25 µL/cm^2^ of silicone oil (viscosity: 100 cSt) was dripped onto the surface of the PEO-treated Al alloy using a micropipette. The silicone oil was uniformly spread over the entire surface within 10 min due to the porous nature of oxide layer formed by PEO. Then, the sample was heat-treated in an electric furnace at 300 °C for 10 min to form a thin PDMS brush, which is SAM, on the surface of the specimen, giving it hydrophobicity and lubrication. The excessive oil layer was gently blown away using compressed air.

A field emission scanning electron microscope (FE-SEM, JSM-7200F, Jeol Inc., Tokyo, Japan) with energy dispersive spectroscopy (EDS) was used to observe the surface and cross-sections and to analyze chemical composition. The crystal structure of oxide layer was analyzed using X-ray diffraction (XRD, SmartLab, Rigaku, Tokyo, Japan) in the range of 2θ between 10° and 80° using Cu target (Kα = 1.5406 Å) under the condition of an acceleration voltage of 45 kV, current of 200 mA, and scanning speed of 3°/min. The hardness of the oxide layer was tested on its cross-section using a Vickers hardness tester under a load of 50 g·f. Fourier-transform infrared spectroscopy (FT-IR, VERTEX 70, Bruker, Billerica, MA, USA) was performed at intervals of 2 cm^−1^ in the range of 499–4000 cm^−1^ to analyze the formation of the PDMS layer on the oxide surface. The wettability and mobility of the water and silicone oil droplets (5 µL) were measured using a contact angle measuring instrument (SmartDrop, Femtobiomed Inc., Seongnam, Republic of Korea) with a digital camera.

Corrosion resistance of surface-treated Al alloy was tested by potentiodynamic polarization and electrochemical impedance spectroscopy (EIS) using a potentiostat (VersaSTAT3, AMETEK, Berwyn, PA, USA) in a 0.1 M HCl solution. Ag/AgCl and Pt mesh were used as the reference and the counter electrode, respectively. Prior to the tests, the sample was immersed in 0.1 M HCl for 20 min to approach a steady-state open circuit potential (OCP). As for the potentiodynamic polarization test, the potential was scanned from −600 to 1000 mV vs. OCP with 2 mV/s of scan rate. As for the EIS measurement, oscillating potentials (10 mV vs. OCP) were applied with a frequency range from 10 kHz to 0.1 Hz. A ball-on-disk type wear machine (Tribometer, J&L Tech, Ansan, Republic of Korea) was used to test the wear resistance. A zirconia (ZrO_2_) ball (diameter: 5 mm) was used as the counter body. And wear load, linear sliding speed, and sliding distance were 2 N, 0.05 m/s, and 10 m, respectively. The coefficient of friction (COF) versus the sliding distance was recorded during each wear test.

## 3. Results and Discussion

The high voltage applied during the PEO treatment of Al alloy causes the dielectric breakdown of the stable passive oxide film of the Al alloy. The dielectric breakdown generates a high temperature spark or arc plasma discharge, so that high temperature oxidation of the base metal and rapid melting and solidification of the oxides occur during the PEO. As a result of these phenomena, the Al surface is covered with crystalline oxides formed at high temperatures.

**Figure 1:**
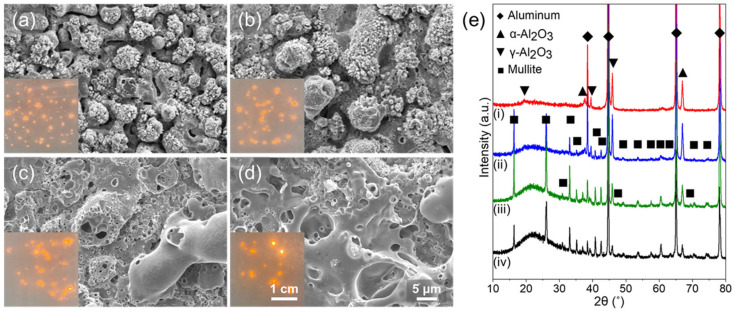
Surface SEM images of porous oxide layer by PEO; (**a**) 10 min, (**b**) 20 min, (**c**) 30 min, and (**d**) 40 min. (**e**) X-ray diffraction (XRD) patterns of porous oxide layer by PEO on Al 6061 alloy; (**i**) 10 min, (**ii**) 20 min, (**iii**) 30 min, and (**iv**) 40 min.

Figure 1 shows the surface images and XRD patterns of oxide layers formed by PEO in Na_2_SiO_3_-based electrolytes under a constant current density of 100 mA/cm^2^, with various processing times. With the increase in PEO processing time, the discharging spots are localized and coarsened (see inserts in Figure 1a–d), which indicate the energy density of PEO increase and higher temperature condition is sustained [43]. The breakdown voltage increases with the thickness of the oxide layer, so that higher electric energy is concentrated to be discharged. Therefore, the localized discharge with higher energy density and temperature due to longer PEO processing time causes bigger pores and a coarser surface structure. Due to fine and dispersed plasma discharge, randomly distributed micro bump structures are formed when PEO is performed for 10 min. With the increase in PEO processing time to 20 min, the plasma discharge was localized, so that the size of micro bump increased. In the case of PEO lasting 30 min, showing more localized plasma discharge, larger micro bumps were formed, and hollow structures caused by the rapid solidification of melted oxides with gas evolution were observed. The micro pumps were rarely observed on the surface of oxide layers formed by PEO lasting 40 min, while the number of hollow structures was increased. In addition, since the localized discharge induces a higher temperature environment on the Al surface, the crystal structure of the oxide layer changed with processing time. In the case of PEO lasting 10 min, the plasma discharge was fine and evenly dispersed, indicating a relatively lower temperature than in other cases, thus only Al oxides (i.e., α-Al_2_O_3_ and γ-Al_2_O_3_) were formed. However, as for PEO for more than 20 min, the more concentrated plasma discharge generates a higher temperature, thus the silica (SiO_2_) formed from silicate can be melted and mixed with Al_2_O_3_. Therefore, due to such concentrated plasma discharge, a crystalline mullite phase (i.e., 3Al_2_O_3_∙2SiO_2_), which can be created at high temperatures, is formed with α-Al_2_O_3_ and γ-Al_2_O_3_ for PEO treatments lasting 20, 30, and 40 min [44].

**Figure 2:**
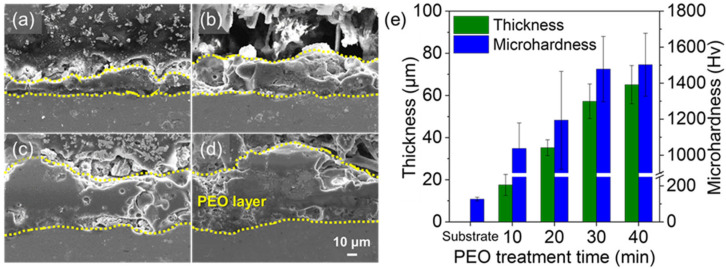
Cross-sectional SEM images of porous oxide layers after PEO; (**a**) 10 min, (**b**) 20 min, (**c**) 30 min, and (**d**) 40 min. (**e**) Thickness and Vickers microhardness of porous oxide layer after PEO.

The change in the plasma discharge with the duration of PEO also affects the thickness and hardness of the oxide layer. Figure 2 shows the cross-section, thickness, and hardness of the oxide layer formed by PEO. With the increase in PEO duration, the region affected by the concentrated plasma discharge increases, so that more oxide is formed and the thickness increases, such as 17 ± 5 μm for 10 min of PEO, 35 ± 4 μm for 20 min of PEO, 57 ± 8 μm for 30 min of PEO, and 65 ± 9 μm for 40 min of PEO. In addition, we estimated the porosity of oxide layers from the cross-sectional SEM images, such as 22% for 10 min, 25% for 20 min, 28% for 30 min, and 30% for 40 min. Regardless of its thickness, since the oxide layer is formed by rapid melting and solidification at high temperature, micropores and cracks due to gas adsorption on molten oxides and rapid cooling of molten oxide are observed in the oxide layer. In addition, the microhardness of the oxide layer increased with PEO duration, such as 1037 ± 142, 1194 ± 272, 1502 ± 175, and 1478 ± 181 Hv for 10, 20, 30, and 40 min of PEO, respectively. The increase in stable crystalline oxide phases with increasing PEO duration enhances the hardness of the oxide layer [45]. The hardness (more than 1000 Hv) of the oxide layers created by PEO is higher than that of general anodic oxide of Al alloys, thus the surface fabricated in this study would have more tolerance to physical impact and abrasion. In addition, since the changes in surface morphology, microstructure, and crystal structure of porous oxide layers by PEO treatment time would also affect the efficiency of silicone oil impregnation on corrosion and wear resistance, we used these four types of PEO layers for silicone oil impregnation.

**Figure 3:**
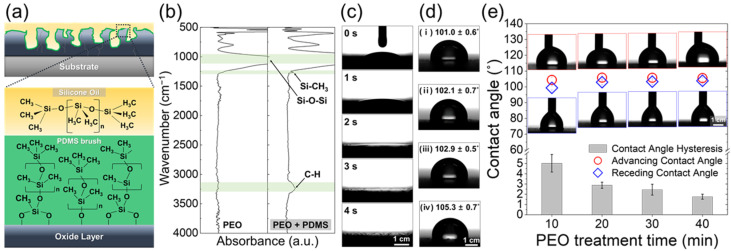
(**a**) Fabrication of PDMS on the porous oxide layer for hydrophobicity and lubrication. (**b**) FT-IR spectra for PEO 30 min and PEO 30 min + PDMS brush. (**c**) Sequential images of silicone oil droplet on the porous oxide layer of PEO 30 min. (**d**) The static contact angle of a water droplet on silicone oil impregnation Al alloy surfaces with PEO; (**i**) 10 min, (**ii**) for 20 min, (**iii**) 30 min, and (**iv**) 40 min. (**e**) Dynamic volumetric contact angle (advancing/receding contact angle and contact angle hysteresis) of a water droplet on Al alloy with PEO + silicone oil impregnation.

Although the oxide layer created by PEO has a significantly high hardness, pores and cracks that provide penetration paths for corrosive media toward the Al substrate are present. Moreover, such a rough surface morphology is vulnerable to contamination. Therefore, it is necessary to employ a post-treatment process that complements these defects. In this study, a silicone oil, in which molecules (PDMS) are able to bond to the oxide surface via simple heat treatment, is impregnated on the porous oxide layer. Since the PDMS bond forming a monomolecular layer on the oxide layer significantly improves its wetting and chemical affinity to silicone oil, the multifunctional lubricant-impregnated surface can be realized (Figure 3a). Moreover, the pores in the oxide layer provide sufficient space for oil retention and also immobilize the oil, preventing drain-out.

In order to identify the PDMS layer on oxide, the silicone-oil-impregnated porous oxide surface with heat treatment was washed three times for 50 min in fresh toluene with ultrasonication, then the surface was analyzed using FT-IR. Peaks related to Si–CH_3_ and C–H at a wavenumber 1261 and 3237 cm^−1^, respectively, indicate the presence of the PDMS layer bound to the oxide (Figure 3b) [46]. Dripped silicone oil droplets on the PEO layer rapidly spread along the surface, indicating that the oxide layer with pores and cracks is suitable for the retention of silicone oil through the formation of a lubricating layer on the oxide surface without the formation of an oil drop (Figure 3c). Moreover, considering the porosity and volume of oxide layers (10 min: 15.3 μL/cm^2^, 20 min: 31.5 μL/cm^2^, 30 min: 51.3 μL/cm^2^, and 40 min: 58.5 μL/cm^2^), 25 μL/cm^2^ of silicone oil fully fills the entire porous structure of oxide layers formed by PEO. Therefore, the lubricating layer with silicone oil is formed on the surface of oxide layer. Due to the PDMS layer on the oxide surface, a slippery lubricant-impregnated surface can be realized, and the surface shows an apparent water droplet contact angle of approximately 100° (Figure 3d), which is generally reported for oil- (or lubricant-)impregnated surfaces. Even though the surface cannot show a significantly high contact angle, since the oil layer inhibits the contact of water droplets to the oxide surface, the surface shows a low contact angle hysteresis of less than 5°, indicating extremely high mobility of the water droplet (Figure 3e). These results imply that the silicone oil impregnation and heat treatment possibly realize the multifunctionalities of slippery lubricant-impregnated surface on the porous oxide layer formed by PEO. In addition, because the rough surface structure of the oxide layer beneath the oil/water interface is effective in reducing contact with water, the contact angle hysteresis of water droplets on silicone-oil-impregnated surfaces decreases with the duration of PEO treatment [47].

**Figure 4:**
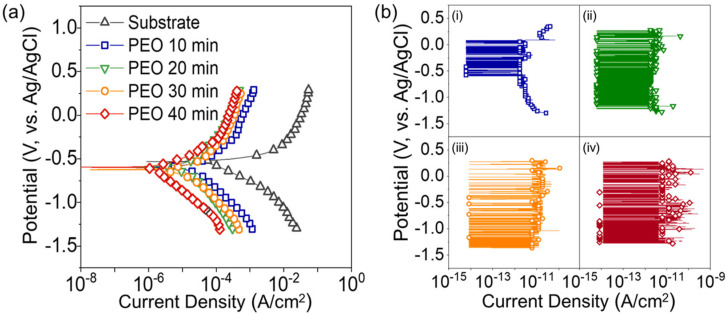
The corrosion resistance of surface-treated Al alloy. (**a**) Potentiodynamic polarization curves of Al alloy with PEO of 10, 20, 30, 40 min. (**b**) Potentiodynamic polarization curves of Al alloy with PEO; (**i**) 10 min, (**ii**) 20 min, (**iii**) 30 min, (**iv**) 40 min + silicone oil impregnation.

Electrochemical potentiodynamic polarization tests were carried out to determine the enhancement of corrosion resistance via silicone oil impregnation, and the results are shown in Figure 4. The oxide layer formed by PEO has significant cracks and pores, which provide a direct path of 0.1 M HCl solution toward the Al substrate. Therefore, the corrosion potential is rarely changed with an increase in the duration of PEO treatment. However, significant changes in the corrosion current density of Al alloy with PEO duration were observed (Figure 4a). The corrosion current density of Al alloy substrate is 7.81 × 10^−5^ A/cm^2^, then it decreases to 2.96 × 10^−5^ A/cm^2^ for 10 min of PEO, 1.61 × 10^−5^ A/cm^2^ for 20 min of PEO, 8.15 × 10^−6^ A/cm^2^ for 30 min of PEO, and 1.25 × 10^−6^ A/cm^2^ for 40 min of PEO. A thicker oxide layer was formed with increasing PEO duration; thus, the active area for corrosion was reduced. Moreover, the length of the penetration path of the corrosive media toward the Al substrate increased concomitantly with the oxide thickness. Nevertheless, because the electrolyte can be sufficiently absorbed into the oxide layer through pores and cracks, the corrosion current density of the Al alloy surface with 40 min PEO treatment was only one order of magnitude lower than that of the bare Al alloy. However, the silicone oil impregnation and heat treatment significantly reduced the corrosion current density of the PEO-treated Al alloys. Corrosive liquids rarely make contact with silicone-oil-impregnated surfaces, and the oxide layers are quite thick, more than 20 μm, resulting in the oxide layer becoming a dielectric layer for electrochemical reactions. Therefore, the response current would be lower than the detection limit of the potentiostat (~6.0 × 10^−12^ A/cm^2^); thus, only noise-like current response is recorded in this study (Figure 4b). However, the corrosion current density is expected to be lower than the measurement limit of this test equipment. Considering that the corrosion current density of an Al alloy substrate is 7.81 × 10^−5^ A/cm^2^, the corrosion current density can be reduced by more than seven orders of magnitude by silicone oil impregnation of the porous oxide layer and its heat treatment.

**Figure 5:**
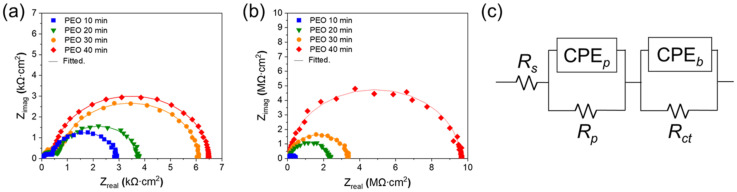
The corrosion resistance of surface-treated Al alloy. EIS Nyquist plot of Al alloy with (**a**) PEO 10, 20, 30, 40 min, and (**b**) PEO 10, 20, 30, 40 min + silicone oil impregnation. (**c**) Equivalent circuit for data fitting.

The potentiodynamic polarization test was not sufficient to obtain a distinguishable difference in oil-impregnated surfaces for corrosion resistance. Therefore, we also used electrochemical impedance spectroscopy (EIS) to evaluate the corrosion resistance of silicone-oil-impregnated surfaces. Figure 5 shows the measurement results of EIS in the Nyquist plot of the PEO-treated samples with or without silicone oil impregnation (Figure 5a,b). In addition, EIS results were fitted using an equivalent circuit (Figure 5c), and the fitted data are listed in Table 1. The *R_s_*, *R_p_*_,_ and the *R_ct_* correspond to the resistance of the electrolyte, the porous thick layer, and the charge transfer reaction at metal/oxide interface. CPE*_p_* and CPE*_b_* represent the capacitance of the double layer on the oxide surface and barrier oxide layer. In addition, *n_p_* and *n_b_* are exponents of CPE*_p_* and CPE*_b_*, respectively. In cases without silicone oil impregnation, the *R_p_* increases with the increase in PEO duration, which causes the formation of a thicker oxide layer. The thicker oxide layer is effective in inhibiting the penetration of corrosive media towards the metal substrate, so the *R_ct_,* indicating the contact area between the corrosive media and metal substrate, is increased with *R_p_*. In the case of PEO for 10 min, the silicone oil impregnation increases the *R_p_* by more than two orders of magnitudes, so the *R_ct_* also increases by more than 26 folds. The efficacy of silicone oil impregnation increases with the thickness of the oxide layer, such as an increase in *R_p_* by more than three orders of magnitudes and an increase in *R_ct_* by more than 470 folds for PEO 40 min with silicone oil impregnation. These results indicate that the silicone-oil-impregnated PEO surfaces have superior corrosion resistance than the wettable hydrophilic porous oxide surfaces. Moreover, it should be noted that the thicker oxide layer with silicone oil impregnation has a higher corrosion resistance.

The internal pores of the oxide layer formed by PEO provides penetration paths for corrosive media to the aluminum substrate. However, the silicone oil immiscible with aqueous solution inhibits the penetration of corrosive media in the porous structure, so that the corrosion resistance is significantly increased via silicone oil impregnation. In addition, since such effect of silicone oil impregnation is strengthened with thickness, the thicker, porous oxide layer impregnated with silicone oil has higher resistance to electrochemical corrosion, implying the better anti-corrosion effects of thick-oil-impregnated layers on Al alloy. The silicone-oil-impregnated porous oxide surface of Al showed an extreme mobility of water droplets (Figure 3e), thus the absorption of water into the porous oxide layer is strongly inhibited. Moreover, the penetration of corrosive media transported with water through pores and cracks is blocked by the silicone oil impregnation of the oxide layer. Enhanced de-wetting to water and aqueous solutions via the silicone oil impregnation of the porous oxide layer and its heat treatment contribute to the significant decrease in corrosion current density and increase in resistance to electrochemical corrosion reaction of PEO-treated Al alloy, indicating that the post-treatment with silicone oil is effective in anti-corrosion.

**Figure 6:**
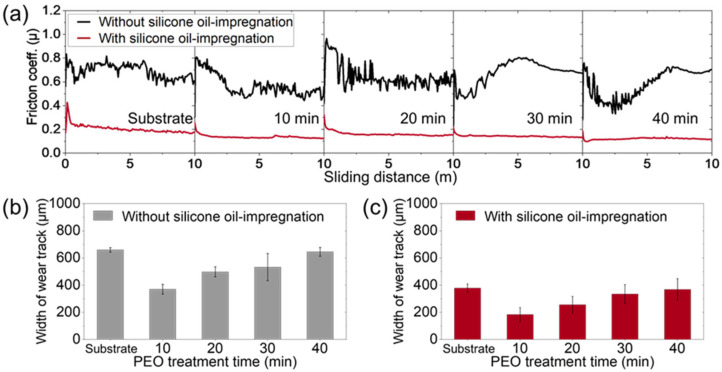
The wear resistance of surface-treated Al alloy. (**a**) Friction coefficient vs. sliding distance without silicone oil impregnation and with silicone oil impregnation. Width of wear track for the PEO-treated samples (**b**) without silicone oil impregnation and (**c**) with silicone oil impregnation.

Wear resistance is one of the important properties of the protective surface treatment of Al alloys. Thus, we tested the wear resistance of the silicone-oil-impregnated surfaces using the ball-on-disk method, and the results are shown in Figure 6. Without the oil impregnation, the coefficient of friction (COF) of the Al alloy substrate and its PEO-treated surface were unstable with the sliding distance of the ball travel (Figure 6a). The average COFs are 0.691 ± 0.060, 0.565 ± 0.091, 0.637 ± 0.094, 0.676 ± 0.109, and 0.580 ± 0.122 for the untreated Al alloy substrate and after 10, 20, 30, and 40 min of PEO treatment, respectively. Such high and unstable COFs indicate the absence of lubrication and that the surface is significantly damaged by wear. Indeed, significant width of wear tracks remained after the wear test, such as 659 ± 17 μm for Al alloy substrate and 334 ± 36, 498 ± 99, 553 ± 37 and 645 ± 32 μm for 10, 20, 30, and 40 min of PEO, respectively (Figure 6b). Due to the hard oxide layer, the wear can be decreased, but the reduction is not significant. In contrast, the silicone-oil-impregnated surfaces showed stable COFs with the distance of ball travel, indicating the lubrication effect of silicone-oil-impregnated oxide layers. The average COFs of silicone-oil-impregnated and heat-treated surfaces were 0.214 ± 0.034 for the Al alloy substrate and 0.137 ± 0.016, 0.161 ± 0.021, 0.143 ± 0.009, and 0.122 ± 0.009 for 10, 20, 30, and 40 min of PEO, which are lower than the un-impregnated samples by more than 65%. In particular, friction on the Al alloy substrate surface can also be decreased by silicone oil impregnation and its heat treatment. Therefore, oil impregnation also decreased the width of wear tracks on the surfaces, such as 378 ± 31 μm for the Al alloy substrate and 183 ± 52, 256 ± 61, 334 ± 70, and 368 ± 79 μm of 10, 20, 30, and 40 min of PEO, respectively (Figure 6c). These results indicate that impregnating silicone oil on the surface of the PEO layer can provide excellent lubrication to the surface of the material exposed to the constant friction environment, thereby significantly enhancing the wear resistance. Meanwhile, although the harder oxide layer is fabricated by the longer PEO duration (Figure 2e), the surface shows wider tracks with wear, regardless of the presence of silicone oil. With increase in PEO duration, the more concentrated discharge is generated to form hollow structures on oxide surface, which can easily be broken by a normal force with a ceramic ball. This broken hollow structure forms hard abrasive particles, enhancing the damage on the surface [48]. Therefore, even with the silicone oil impregnation, more precise control of the duration of PEO treatment, which is a key factor affecting the hardness and crystal structure (Figure 1e), is required to maximize the wear resistance of the porous oxide layer formed via PEO.

**Figure 7:**
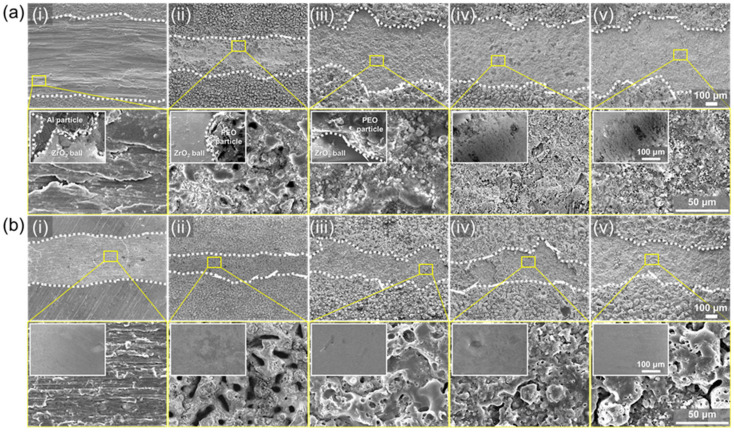
The SEM images of wear track. (**a**) Samples without silicone oil impregnation. (**b**) Samples with silicone oil impregnation; (**i**) Substrate, (**ii**) PEO 10 min, (**iii**) PEO 20 min, (**iv**) PEO 30 min, and (**v**) PEO 40 min. And insert images are the SEM images of worn scars of the counter body for each wear test.

The surfaces of worn oxide and counter body (ZrO_2_ ball) were also observed to analyze changes of wear mechanism via silicone oil impregnation (Figure 7). Without oil impregnation, the Al substrate shows the deformation and delamination of worn surfaces, with Al particle debris on counter body indicating adhesive wear (Figure 7a). In contrast, the worn track of PEO-treated Al surfaces for 10 and 20 min shows the blocky debris filled in the pores and adhered on counter body, indicating abrasive wear. In the case of PEO for 30 and 40 min, debris filled in the pores of the wear track can be also found, but the counter body was also significantly worn. The broken hollow structure formed at high temperature of PEO for 30 and 40 min contributes to the formation of hard abrasive particles during the wear test. In addition, the hardness of the oxide layer formed by PEO for 30 and 40 min is similar or higher than the hardness of ZrO_2_ ball (~1400 Hv) [49,50,51]. Therefore, the counter body (i.e., ZrO_2_ ball) can be worn on the oxide surfaces formed via PEO for 30 and 40 min, thereby the worn surface shows wide wear track while the surface porous oxide still remained. Silicone oil impregnation reduces the wear width of bare Al substrate, but the worn surface still shows the adhesive wear (Figure 7b). In the case of oxide surfaces with silicone oil impregnation, the width of wear track significantly decreased, and abrasive debris was not found in the pores and on the counter body. Moreover, worn damages were not found on the ZrO_2_ counter body. These results indicate that the silicone oil impregnation reducing the friction coefficient inhibits the formation of hard abrasive debris, so that the worn damage on the oxide surface, as well as the counter body, is impeded.

**Figure 8:**
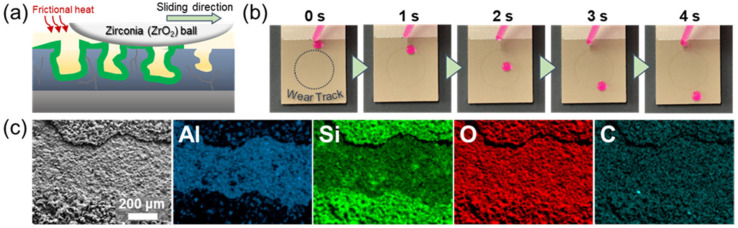
Self-healing of PDMS layer on Al alloy with PEO 30 min + silicone oil impregnation. (**a**) Cross-sectional schematic of the formation of PDMS brush on the wear track of the PEO layer. (**b**) The movement of water droplets across the wear track. (**c**) SEM and EDS mapping images on the wear track.

Physical damage to de-wetting (i.e., hydrophobic) surfaces is the most serious challenge preventing practical applications. In particular, the physical impact on de-wetting surfaces not only removes the hydrophobic functional layer but also destroys the porous surface structure [52]. However, the PDMS bonding on the oxide surface can be regenerated as long as the silicone oil remains impregnated within the porous structure, and the slippery lubricant-impregnated surface does not require a precisely controlled surface structure. Therefore, the property of the silicone-oil-impregnated surface can be restored. To demonstrate the self-healing capability of silicone-oil-impregnated surfaces, a wear track with a width of 350 μm was made on the sample surface using the ball-on-disk method with 3 N of wear load, 0.05 m/s of linear speed, and 5 m of sliding distance. After removing the silicone oil from the sample by washing it in toluene three times for 50 min, distilled water stained with Rhodamine B was dropped on the sample inclined at 25°. During the wear test, the counter body damages the oxide surface with a PDMS layer, which is formed via silicone oil impregnation and post heat treatment. Thus, the hydrophobicity of the wear track can be eliminated. However, the friction between the ZrO_2_ ball and the porous oxide layer generates a frictional heat, which promotes the silane bonding between PDMS and oxide surface similar to the post heat treatment so that the PDMS monolayer can be recovered (Figure 8a). Thus, even though the surface of the wear track is damaged, silicone oil is retained within the hydrophobized porous oxide structure, and the water droplet is not pinned on the wear track but slides along the inclined surface across the wear track with a width of 600 μm (Figure 8b). Moreover, EDS mapping revealed a considerable content of carbon on the wear track of the tested sample after removing the silicone oil (Figure 8c), indicating the reformation (i.e., self-healing) of a monolayer of PDMS on the wear track. These results also indicate that the de-wetting property of the damaged region on the silicone-oil-impregnated porous oxide surface can be healed via simple heat treatment [53]. From these results, it is found that the Al alloy with porous oxide layer due to PEO being impregnated with silicone oil has a self-healing capability to recover de-wetting property even under external physical damage, such as wear and abrasion.

Silicone oil impregnation of porous anodic oxide of Al alloy formed by PEO not only improves the corrosion resistance but also suppresses the destructive abrasive wear. Moreover, the de-wetting of silicone-oil impregnated surface can be healed so that the surface shows a corrosion tolerance to physical damage. These advantages of silicone-oil-impregnated hard porous oxide would enable Al alloys to be applied to various engineering fields requiring high hardness, corrosion resistance, and wear resistance, such as the aerospace industry (e.g., valve bodies, actuators, and vanes), automotive engines (e.g., pistons and cylinder liners and bodies), gas/oil extraction (e.g., seals, rings and valves), and textile processing (rotors, rollers, and shafts) [54,55,56]. Therefore, silicone oil impregnation can be a promising candidate for the post-treatment of plasma electrolytic oxidized Al alloy, extending the application fields by improving the corrosion and wear resistance.

## 4. Conclusions

Herein, we investigated lubricant impregnation as a post-treatment to PEO of an Al alloy. Silicone oil impregnation of the hard porous oxide layer resulting from PEO treatment and its heat treatment creates PDMS-SAM on the oxide surface, creating a water-repellent surface with significantly enhanced mobility of water droplets on the Al alloy surface. Such water repellency inhibits the absorption and penetration of corrosive media via pores and cracks, which are critical defects of oxide layers formed via PEO; thereby, the corrosion resistance of the PEO-treated Al alloy significantly improved through silicone oil impregnation and its heat treatment. The silicone oil within the porous oxide layer provides a lubrication effect under wear and abrasion conditions; thus, the surface has a significantly enhanced wear resistance. Moreover, because the PDMS monolayer can be regenerated on the surface of an oxide, the silicone-oil-impregnated porous oxide surface is tolerant to physical damage. The approach demonstrated in this study would be an effective candidate to complement defects of Al PEO by improving the corrosion and wear resistance with self-healing abilities.

## Figures and Tables

**Table 1:** Fitted data for parameter of equivalent circuit.

Sample Name	Silicone Oil-Impregnation	CPE*_p_*, nF/cm^2^	*n_p_*	*R_p_*, Ω cm^2^	CPE*_b_*, nF/cm^2^	*n_b_*	*R_ct_*, Ω cm^2^
PEO 10 min	X	359.00	0.98	2481	2393	0.97	278.3
PEO 20 min	X	103.00	0.97	3427	928.2	0.99	351.3
PEO 30 min	X	45.44	0.99	3600	780.5	0.98	400.9
PEO 40 min	X	35.96	0.98	6010	562.0	0.99	600.3
PEO 10 min	O	1.44	0.97	417,300	9.54	0.98	72,890
PEO 20 min	O	0.88	0.97	2,186,000	4.49	0.99	90,600
PEO 30 min	O	0.53	0.98	3,265,000	2.71	0.97	115,600
PEO 40 min	O	0.24	0.96	9,536,000	0.95	0.98	282,000

## Data Availability

Not applicable.
